# Drosophila Longevity Assurance Conferred by Reduced Insulin Receptor Substrate *Chico* Partially Requires *d4eBP*

**DOI:** 10.1371/journal.pone.0134415

**Published:** 2015-08-07

**Authors:** Hua Bai, Stephanie Post, Ping Kang, Marc Tatar

**Affiliations:** Department of Ecology and Evolutionary Biology, Division of Biology and Medicine, Brown University, Providence, Rhode Island, United States of America; The Scripps Research Institute, Scripps Florida, UNITED STATES

## Abstract

Mutations of the insulin/IGF signaling (IIS) pathway extend Drosophila lifespan. Based on genetic epistasis analyses, this longevity assurance is attributed to downstream effects of the FOXO transcription factor. However, as reported FOXO accounts for only a portion of the observed longevity benefit, suggesting there are additional outputs of IIS to mediate aging. One candidate is target of rapamycin complex 1 (TORC1). Reduced TORC1 activity is reported to slow aging, whereas reduced IIS is reported to repress TORC1 activity. The eukaryotic translation initiation factor 4E binding protein (4E-BP) is repressed by TORC1, and activated 4E-BP is reported to increase Drosophila lifespan. Here we use genetic epistasis analyses to test whether longevity assurance mutants of *chico*, the Drosophila insulin receptor substrate homolog, require Drosophila *d4eBP* to slow aging. In *chico* heterozygotes, which are robustly long-lived, *d4eBP* is required but not sufficient to slow aging. Remarkably, *d4eBP* is not required or sufficient for *chico* homozygotes to extend longevity. Likewise, *chico* heterozygote females partially require *d4eBP* to preserve age-dependent locomotion, and both *chico* genotypes require *d4eBP* to improve stress-resistance. Reproduction and most measures of growth affected by either *chico* genotype are always independent of *d4eBP*. In females, *chico* heterozygotes paradoxically produce more rather than less phosphorylated 4E-BP (p4E-BP). Altered IRS function within the IIS pathway of Drosophila appears to have partial, conditional capacity to regulate aging through an unconventional interaction with 4E-BP.

## Introduction

Mutations of the insulin/IGF-signaling (IIS) pathway in Drosophila and *C*. *elegans* slow aging when measured by adult survival and by the decline of age-associated traits [1–7]. In Drosophila, as in mammals, the insulin-like receptor is auto-phosphorylated upon ligand binding and subsequently recruits and phosphorylates the SHB2 domain-containing insulin receptor substrate (CHICO, the IRS1-4 homolog) [8–10]. Activated Drosophila IRS/CHICO transduces signaling through phosphatidylinositol-3-OH kinase PI(3)K which then regulates effector pathways including PKB/ AKT, Tsc2, PRAS40 and GSK [11–13]. Among these, AKT phosphorylates and represses FOXO. Genetic epistasis studies in worm and fly have examined whether the FOXO transcription factor is required for reduced IIS signaling to extend lifespan. In *C*. *elegans*, longevity assurance conferred by mutations of *daf-2*, the Insulin/IGF receptor ortholog, requires FOXO encoding *daf-16* to extend lifespan [3,14,15]. Similarly, *dfoxo* is required for reduced IIS in Drosophila to extend life span [16,17]. But notably, null mutations of *dfoxo* in Drosophila only partially restore lifespan of IIS mutants toward wildtype. These observations suggest that IIS potentially signals through additional, unexplored factors to control aging.

One candidate involves target of rapamycin (TOR). PI3K/AKT can modulate TOR activity in mammalian and Drosophila cell culture [18,19], although the interaction of PI3K/AKT and TOR activity in Drosophila tissue is less clear [12,20]. In the standard view, activated AKT blocks the repressive effects of TSC1/2 upon target of rapamycin complex 1 (TORC1), permitting TORC1 to be autonomously activated by amino acids. TORC1 stimulates numerous growth-associated systems including protein translation machinery by phosphorylating the S6 ribosomal kinase (S6K) and the translation initiation factor inhibitory protein 4E-BP. PI3K/AKT also regulates TORC1 through PRAS40 (proline-rich Akt substrate of 40 kDa). AKT phosphorylates PRAS40 and thus reduces its ability to bind and inhibit TORC1 in cell culture and in Drosophila ovaries, but not so in somatic tissue [12].

Drosophila TOR and 4E-BP (encoded by *thor*, also denoted *d4eBP*) were seen to regulate aging in a number of studies. Lifespan was extended when TOR signaling was inhibited by over-expressing genes of the tuberous sclerosis complex (*dTsc1*, *dTsc2*), and by expressing dominant-negative forms of TOR and S6K [21]. The heteroallelic *dTOR* ^*7/P*^ mutant genotype extended lifespan 20% and prevented age-dependent decline in cardiac performance [22]. Drosophila 4E-BP overexpressed in a constitutively activated form extended lifespan in adults fed a rich diet [23]. Expression of constitutively active 4E-BP in muscle is sufficient to extend lifespan and preserve age-associated muscle function, potentially because this systemically reduces circulating insulin-like peptides [24]. Likewise, ectopic expression of *d4eBP* specifically within the fly heart protects against age-related decline in myocardial function and cardiac stress resistance [25]. Finally, rapamycin increases adult Drosophila survival, although not in every reported case, presumably because this drug inhibits TOR [26–28]. Together these studies suggest that reduced TORC1 activity in Drosophila slows or retards aging, and that repression of 4E-BP might play a role in this process [29].

Based on these observations, it is natural to propose that reduced IIS modulates aging not just through activating FOXO but also by increasing the translation-inhibitory action of 4E-BP. Here we test this hypothesis by genetic epistasis analysis between mutants of *chico* (encoding the IRS1-4 homolog) and of *d4eBP/thor*. If reduced IIS increases 4E-BP activity (reduces p4E-BP) to affect longevity assurance, we expect the lifespan of *chico d4eBP* double mutants to be restored toward that of wildtype.

Homozygote and heterozygote genotypes of the *chico* ^*1*^ allele robustly extend Drosophila lifespan [2,30]. The *chico* ^*1*^ allele is a P-element *P{ry11}* insertion located in the insulin receptor substrate pleckstrin homology domain of CHICO [8,9,31]. Genetic analysis indicates *chico* ^*1*^ is a loss of function mutation [31]. Homozygotes of *chico* ^*1*^ (*ch* ^*-/-*^) have been studied for how they affect growth, insulin/IGF signaling, development, oogenesis, and physiology [12,32]. Although the impact of *chico* ^*1*^ heterozygotes (*ch* ^*+/-*^) remain relatively less explored, *ch* ^*-/-*^ and *ch* ^*+/-*^ produce striking differences despite their similar ability to extend lifespan: *ch* ^*-/-*^ are small, sterile and develop slowly while *ch* ^*+/-*^ are fecund, and have normal size and development time [2,31]. To facilitate analyses of *chico* ^*1*^ in aging, Tu et al. [30] crossed this allele into a *cinnabar*; *rosy* genetic background (*cn* ^*1*^/*cn* ^*1*^; *ry* ^*506*^/*ry* ^*506*^) so that stocks could be perpetually maintained by breading heterozygotes, and sibs would segregate all *chico* genotypes in a coisogenic background each generation (Among sibs: Heterozygotes are normal size and cinnabar eyes. Wildtype are normal size, apricot eyes by virtue of both *cn* and *ry*. Homozygotes are dwarf with cinnabar eyes).

Drosophila 4E-BP is encoded by *d4eBP* (also known as *thor*). This locus was originally identified as a *P{lacW}* insertion (*thor* ^*1*^) with defective innate immune function, and was proposed to produce 4E binding protein based on peptide homology [33,34]. P-element excision was used to produce a null allele (*thor* ^*2*^) by deleting ~1600 nucleotides in the regulatory and coding region, and to generate a perfect excision-revertant wildtype strain *thor* ^*1rv1*^ [33]. Although overexpression of d4E-BP antagonizes cell growth (wing discs, eyes) in response to PI(3)K/Akt signaling during development [19], homozygotes of *thor* ^*2*^ are largely viable, fertile and normal sized [35,36]. Phenotypes are most apparent under conditions of stress, where mutants show reduced resistance to hydrogen peroxide, and increased mortality during fasting that is associated with elevated rates of fat catabolism [35,37].

## Materials and Methods

### Fly husbandry

Flies were reared and maintained at 25°C, 40% relative humidity and 12-hour light/dark cycle. Standard cornmeal-sugar-yeast diet was used in all experiments (23 g/L SAF yeast (Lesaffre yeast corporation, Milwaukee, WI, USA), 100 g/L sucrose, 47 g/L cornmeal and 7 g/L agar, 0.2% Tegosep (methyl4-hydroxybenzoate, Sigma, St. Louis, MO, USA)).

### Generation of *d4eBP*, *chico* double mutants

Drosophila *d4eBP* null mutants (*Thor* ^*2*^) were obtained from Dr. Deborah A. Kimbrell [33]. The *chico* mutant stock (*y* ^*1*^ *; chico* ^*1*^ *cn* ^*1*^ *; ry* ^*506*^) was previously made in our laboratory, and has been perpetually maintained by breeding heterozygotes [17,30]. Both *chico* and *d4eBP* are on the second chromosome (arm 2L), therefore, to introduce the *d4eBP* mutation into the *chico*-segregation stock we first backcrossed and recombined *Thor* ^*2*^ to the genotype *y* ^*1*^ *; cn* ^*1*^ *; ry* ^*506*^ to produce *y* ^*1*^ *; Thor* ^*2*^ *cn* ^*1*^ *; ry* ^*506*^. Subsequently, *d4eBP*, *chico* double mutants (*y* ^*1*^ *; Thor* ^*2*^ *chico* ^*1*^ *cn* ^*1*^ *; ry* ^*506*^) were produced through recombination between *y* ^*1*^ *; chico* ^*1*^ *cn* ^*1*^ *; ry* ^*506*^ and *y* ^*1*^ *; Thor* ^*2*^ *cn* ^*1*^ *; ry* ^*506*^. See supporting information (S1 Text and S1, S2 and S3 Figs) for details of genetic design.

### Demography and survival analysis

Newly enclosed flies were allowed to mate for two days, then separated by sex and genotype, and assigned to replicate one-liter demography cages at a density of 125 flies per cage. Three independent cages were set-up per genotype. Two independent demography trials were performed and all cohorts within a trial were studied concurrently. Food was changed every two days, at which time dead flies were removed from the cage and counted. Survival analysis was conducted with JMP statistical software (SAS Institute, Cary, NC, USA), and data from replicate cages were combined. Mortality distributions were compared by Log-Rank and proportional hazard analyses.

### Climbing

Climbing ability (negative geotaxis assay) was measured by tapping flies to the bottom of an empty vial and counting (with video recording) flies that climbed 8 vertical centimeters within 20 seconds. Forty females (10 per vial) were scored once a week for each genotype.

### Fecundity

Females from each genotype were collected one day after eclosion and mated with wildtype males. Two days after mating, females were separated and kept in vials with standard fly food (ten vials per genotype, two females per vial). Over the following 10 days (beginning as females were 3 d old), females were passed to new vials daily, and eggs were counted.

### Stress resistance

Starvation resistance was tested in 3-day-old female and male adults. Flies were transferred into glass vials containing 0.8% agar in PBS; dead flies were counted twice a day. Resistance to oxidative stress was tested in 3-day-old flies previously maintained on standard food then transferred into glass vials containing 1% agar, 5% sugar and 20mM paraquat (Sigma, St. Louis, MO, USA). Dead flies were counted twice a day. For both assays, a total of 50 flies were used for each genotype (10 flies per vial). Survival differences were analyzed by proportional hazard analysis.

### Body weight and wing area

Eggs laid upon grape juice plates by 30 females were collected within a four-hour interval to measure larval development rate. Fifty eggs were transferred to a standard food vial; time to adult eclosion was recorded daily. Net adult body weight was measured from a pool of 10 flies, beginning three days after their eclosion from the controlled density vials, with four replicates per genotype and sex. From these same adults, total wing blade area was measured from images of slide-mounted tissue using ImageJ (imagej.nih.gov/ij). Fifteen flies were measured for each genotype, each sex.

### Western blot

Antibodies for Akt (#9272), phospho-Akt (Ser505) (#4054), non-phospho-4E-BP1 (Thr46) (#4923), phospho-4E-BP1 (Thr37/46) (#2855) and β-actin (#4967) were purchased from Cell Signaling Technology (Danvers, MA, USA). Ten flies were homogenized in 100 μl RIPA buffer with protease inhibitor cocktail (Sigma, St. Louis, MO, USA) and PhosSTOP phosphatase inhibitor cocktail (Roche, Nutley, NJ, USA). Supernatant was incubated with LDS loading buffer (Invitrogen, Grand Island, NY, USA) at 70°C for 10 min. Thirty micrograms of denatured protein was separated on 10% NuPAGE Novex 4–12% Bis-Tris precast polyacrylamide gels (Invitrogen, Grand Island, NY, USA) and transferred to PVDF membranes. Following incubation with primary and secondary antibodies, blots were visualized with Pierce ECL western blotting substrate (Thermo Fisher Scientific, Waltham, MA, USA). Three independent biological replicates were generated for all analyses, and samples for AKT and 4E-BP produced at different times. Band intensity was quantified with Image Lab software (Bio-Rad, Hercules, CA, USA).

## Results

### Aging and reproduction

**Fig 1:**
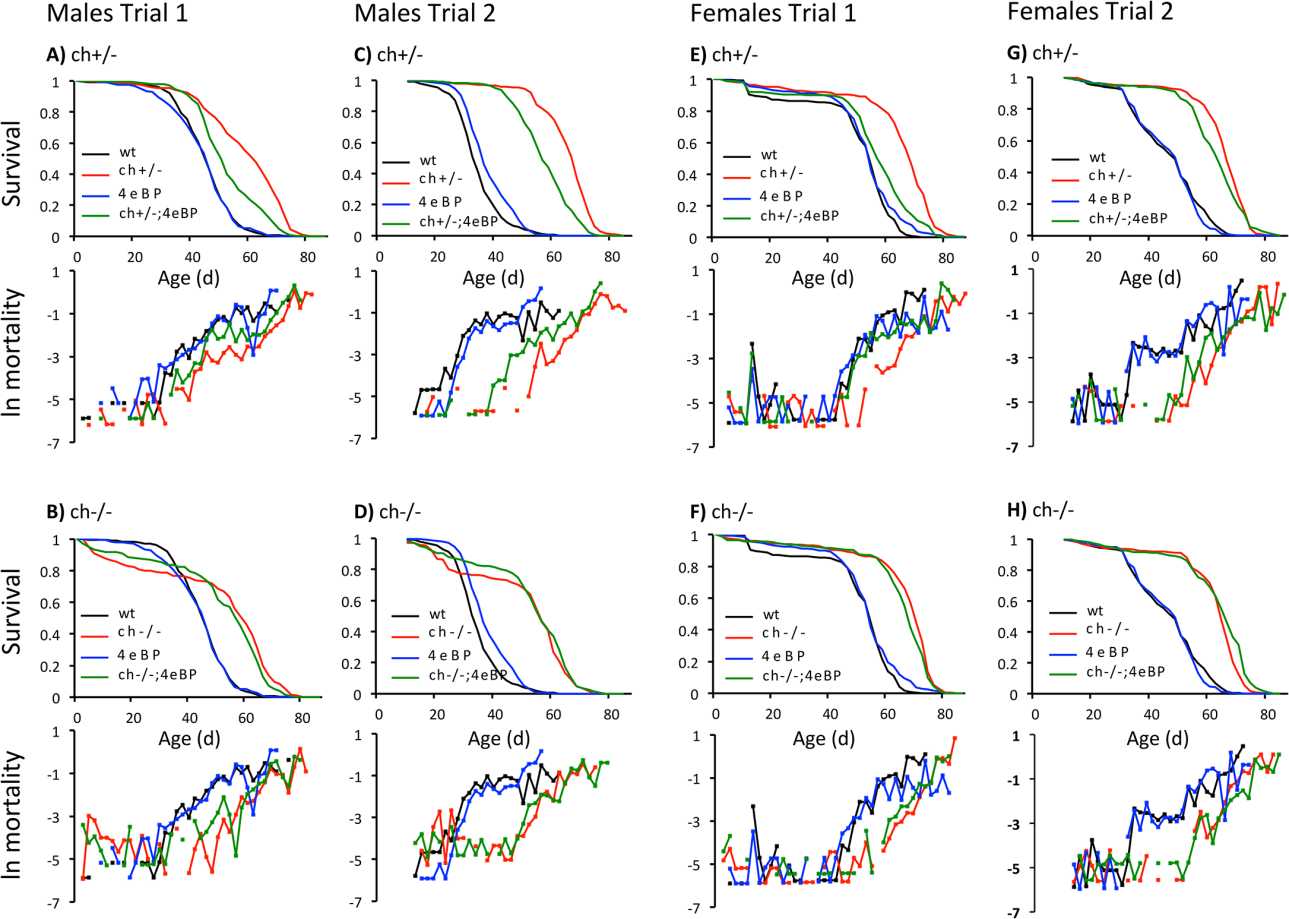
Survivorship and mortality of male and female adult Drosophila with single and combined mutations of *chico* and *d4eBP*. Cohorts of all genotypes were aged concurrently in two independent trials. Deaths in Trial 2 were recorded beginning at 10 days of age. Mortality rate is plotted as ln(*μ*_*x*_), estimated as ln(-ln(1-*q*_*x*_)) where *q*_*x*_ is age-specific mortality. Panels **A**, **C**, **E** and **G** plot *chico* heterozygotes *ch* ^*+/-*^ relative to wildtype, *d4eBP* null mutant and the double mutant *ch* ^*+/-*^ *d4eBP*. Panels **B**, **D**, **F** and **H** plot *chico* homozygotes *ch* ^*-/-*^ relative to wildtype, *d4eBP* null mutant and the double mutant *ch* ^*-/-*^ *d4eBP*.

Life tables from single sex cohorts produced consistent outcomes in two independent demography trials. Adult survival of *ch* ^+/-^ (*chico* ^+^/*chico* ^1^) was robustly increased as a result of uniformly reduced age-specific mortality (Fig 1A, 1C, 1E and 1G). The *ch* ^+/-^ genotype reduced mortality approximately 4-fold relative to wildtype (Table 1). The double mutant *ch* ^+/-^ *d4eBP* partially ameliorated this longevity assurance by increasing age specific mortality relative to *ch* ^+/-^ about 2-fold in three cases and 18% in one case (Table 1). The *d4eBP* mutation produced little or no effect on mortality relative to wildtype, indicating that loss of *d4eBP* in the double mutant reduces longevity assurance of this genotype through genetic interactions (epistasis) rather than by independent effects. Together these data demonstrate that longevity assurance conferred by *ch* ^+/-^ requires 4E-BP to some extent in both males and females.

**Table 1:** Life table and proportional hazard survival analysis statistics of adult Drosophila wildtype, *chico*, *d4eBP* and *chico d4eBP* genotypes. Independent replicate trials, sexes (males & once mated females) maintained as separate cohorts. Number: adults for combined cages of synchronous cohorts. Upper (UL) and lower (LL) 95% confidence intervals for median lifespan. Relative risk estimated from Cox proportional hazard analyses for each genotype (row) relative to *chico* genotype (column); probability > χ ^2^ based on log-likelihood (***) for p < 0.0001. Relative risk indicates fold change of the row genotype relative to the column *chico* genotype. Relative risk less than one (significant estimates in italics) indicate reduced mortality relative to the column *chico* genotype. Relative risk greater than one (significant estimates underlined) indicates increased mortality relative to the column *chico* genotype.

**Trial 1**	**Males**						
					*Relative risk with respect to chico genotype*:		
**Genotype**	**Number**	**Mean (d)**	**Median (d)**	**(LL, UL)**	**wildtype**	***ch*** ^***+/-***^	***ch*** ^***-/-***^
wildtype	356	44.8	46	(44, 46)			
*d4eBP*	481	43.8	46	(44, 46)	**1.03 (0.730)**		
*ch* ^*+/-*^	361	58.7	62	(60, 64)	***0*.*264*** (***)		
*ch* ^*-/-*^	367	50.8	60	(58, 62)	***0*.*331*** (***)		
*ch* ^*+/-*^ *d4eBP*	250	52.2	52	(50, 52)	***0*.*481*** (***)	**1.84** (***)	
*ch* ^*-/-*^ *d4eBP*	356	51.2	58	(56, 60)	***0*.*403*** (***)		**1.22 (0.0167)**
**Trial 1**	**Females**						
					*Relative risk with respect to chico genotype*:		
**Genotype**	**Number**	**Mean (d)**	**Median (d)**	**(LL, UL)**	**wildtype**	***ch*** ^***+/-***^	***ch*** ^***-/-***^
wildtype	370	49.4	54	(54, 56)			
*d4eBP*	370	52.3	54	(54, 54)	***0*.*684*** (***)		
*ch* ^*+/-*^	457	64	68	(68, 70)	***0*.*228*** (***)		
*ch* ^*-/-*^	373	64.7	70	(70, 70)	***0*.*213*** (***)		
*ch* ^*+/-*^ *d4eBP*	383	54.8	56	(56, 58)	***0*.*486*** (***)	**2.12** (***)	
*ch* ^*-/-*^ *d4eBP*	251	63.2	68	(66, 68)	***0*.*250*** (***)		**1.17 (0.0501)**
**Trial 2**	**Males**						
					*Relative risk with respect to chico genotype*:		
**Genotype**	**Number**	**Mean (d)**	**Median (d)**	**(LL, UL)**	**wildtype**	***ch*** ^***+/-***^	***ch*** ^***-/-***^
wildtype	328	34.1	34	(32, 34)			
*d4eBP*	375	37.7	36	(36, 38)	***0*.*759 (0*.*003)***		
*ch* ^*+/-*^	304	64.6	68	(66, 68)	***0*.*0516*** (***)		
*ch* ^*-/-*^	204	49.8	56	(54, 58)	***0*.*139*** (***)		
*ch* ^*+/-*^ *d4eBP*	357	56.8	56	(56, 58)	***0*.*108*** (***)	**2.09** (***)	
*ch* ^*-/-*^ *d4eBP*	140	52.3	56	(54, 58)	***0*.*123*** (***)		**0.884 (0.261)**
**Trial 2**	**Females**						
					*Relative risk with respect to chico genotype*:		
**Genotype**	**Number**	**Mean (d)**	**Median (d)**	**(LL, UL)**	**wildtype**	***ch*** ^***+/-***^	***ch*** ^***-/-***^
wildtype	353	45.7	48	(44, 50)			
*d4eBP*	392	45.6	48	(46, 50)	**1.12 (0.113)**		
*ch* ^*+/-*^	368	63.9	66	(66, 68)	***0*.*181*** (***)		
*ch* ^*-/-*^	284	61.2	64	(64, 66)	***0*.*237*** (***)		
*ch* ^*+/-*^ *d4eBP*	351	61	64	(62, 64)	***0*.*212*** (***)	**1.18 (0.0315)**	
*ch* ^*-/-*^ *d4eBP*	266	62.4	66	(64, 68)	***0*.*182*** (***)		***0*.*768 (0*.*002)***

This demographic outcome for *ch* ^+/-^ contrasts to results from *chico* homozygotes (*ch* ^-/-^, *chico* ^1^/*chico* ^1^) (Fig 1B, 1D, 1F, and 1H). As with *chico* heterozygotes, *ch* ^-/-^ consistently increases survival relative to wildtype and *d4eBP* by uniformly reducing age-specific mortality approximately 4-fold (Table 1). Remarkably, there is no appreciable difference in survival between *ch* ^-/-^ and the double mutant *ch* ^-/-^ *d4eBP*. 4E-BP is not required to realize longevity assurance of the *ch* ^-/-^ genotype.

**Fig 2:**
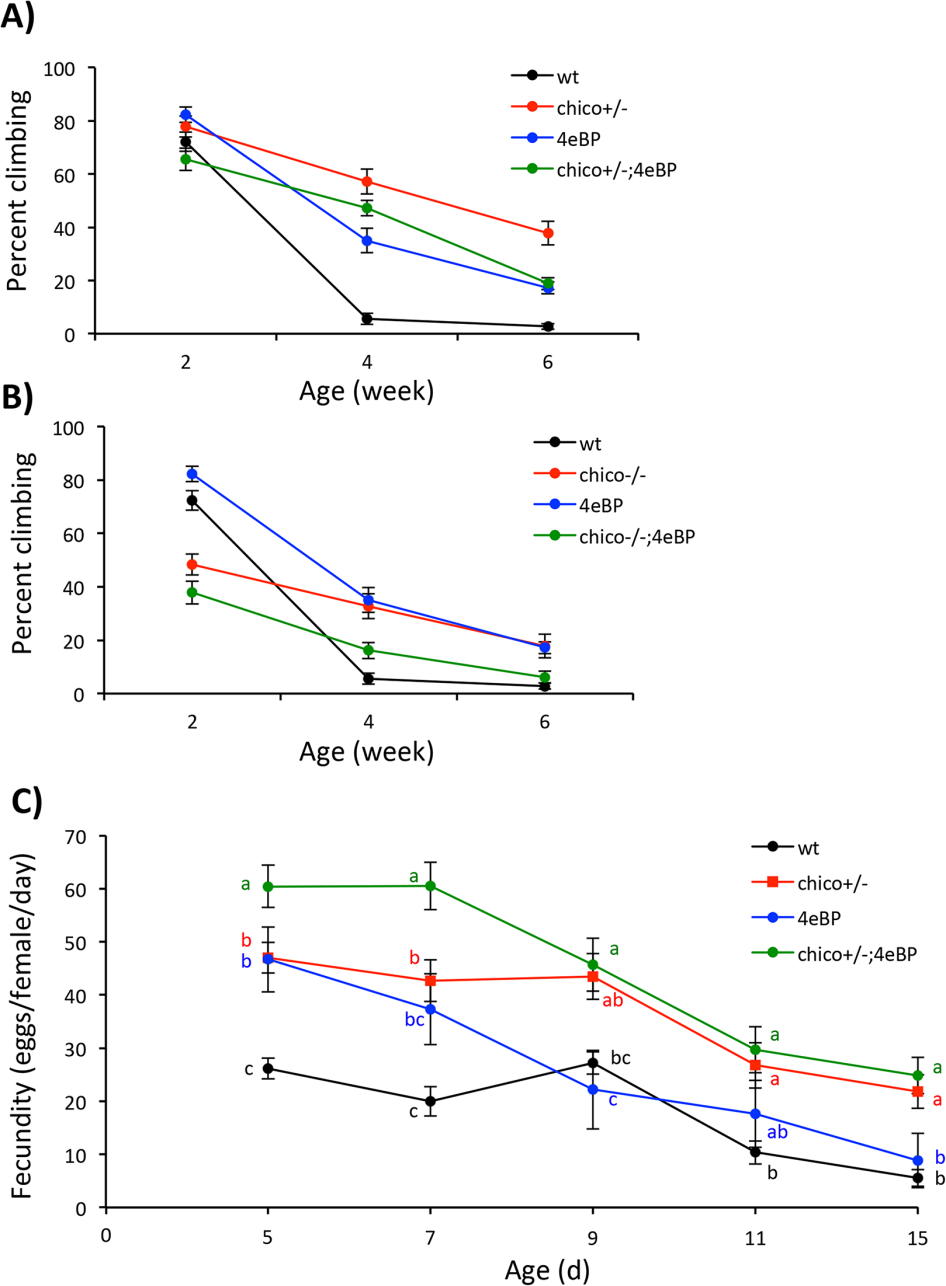
Age dependent decline in climbing and egg production of Drosophila with single and combined mutations of *chico* and *d4eBP*. Vertical climbing was measured from cohorts simultaneously aged to three time points (2, 4, 6 weeks). **A)** Percent climbing for *chico* heterozygotes *ch* ^*+/-*^ relative to wildtype, *d4eBP* null mutant and the double mutant *ch* ^*+/-*^ *d4eBP*. **B)** Percent climbing for *chico* homozygotes *ch* ^*-/-*^ relative to wildtype, *d4eBP* null mutant and the double mutant ch^-/-^ *d4eBP*. **C)** Per capita egg production (with s.e. means) at ages five through 13 days old for *chico* heterozygotes *ch* ^*+/-*^ relative to wildtype, *d4eBP* null mutant and the double mutant *ch* ^*+/-*^ *d4eBP*. Within each age, differences in egg production evaluated by ANOVA with Tukey post-hoc test to resolve means that differ significantly, indicated by discordant letters.

Aside from survival, decline in performance with age is a central feature of animal senescence [38,39]. The percentage of wildtype flies able to adeptly climb declines from about 70% at two weeks old to less than 10% by four weeks old (Fig 2). *Chico* heterozyogotes are robust climbers at two weeks and loose this ability only at a slow rate through age six weeks (Fig 2A). From weeks two to four the climbing index of *ch* ^+/-^ *d4eBP* declines at the same rate as *ch* ^+/-^ but with slightly lower overall performance. Statistical analysis for an effect of *d4eBP* upon the rate of change in the *ch* ^*+/-*^ background from weeks two to four indicates there is no significant interaction between these genes as they affect decline in climbing (general linear model with log likelihood ratio test, β_genotype x age(2–4)_ = 4.17, χ^2^ = 1.01, p = 0.32); there is no genetic epistasis in this time frame. Across weeks four and six there is significant genetic interaction; the double mutant looses climbing ability faster than predicted by the *ch* ^+/-^ mutant alone (β_genotype x age(4–6)_ = -8.32, χ^2^ = 4.53, p = 0.033). At least in one age category, *ch* ^+/-^ appears to partially require *d4eBP* to retard functional senescence.

Similar analyses with *ch* ^-/-^ did not reveal any genetic interaction with *d4eBP* (Fig 2B). While the *ch* ^-/-^ females were relatively weak climbers at young ages, they loose this capacity slowly as they age. Although starting at a low ability at young age, the rate of decline in the double mutant is similar to *ch* ^-/-^ (β_genotype x age(2–4)_ = -3.06, χ^2^ = 0.367, p = 0.544; β_genotype x age(4–6)_ = 2.50, χ^2^ = 0.479, p = 0.489) As with demographic aging there is no evidence that retarded functional aging conferred by *ch* ^-/-^ requires 4E-BP.

Egg production declines with age in mated female Drosophila (Fig 2C). From age 5 to 13 days old, wildtype fecundity declined from about 25–30 eggs per female per day to about 5–10 eggs per day. *Chico* heterozyogotes are more fecund than wildtype even though they are longer-lived (note: *ch* ^-/-^ are sterile). The rate of decline in egg production is similar for *ch* ^+/-^ and wildtype, suggesting that reproductive aging is not retarded by *ch* ^+/-^. The interaction between *ch* ^+/-^ and *d4eBP* for egg production is complex. The genotypes have additive effects at days five and seven where the double mutant is more fecund than each single genotype. By day nine and thereafter, the fecundity of *d4eBP* is similar to that of wildtype while the double mutant is similar to that of *ch* ^+/-^. At these later ages the *d4eBP* mutation no longer contributes to fecundity in the double mutant. Loss of 4E-BP generates extra fecundity only in young females and this is additive to the impact of *ch* ^+/-^. Overall, *ch* ^+/-^ does not retard reproductive aging or interact genetically with *d4eBP* to affect egg production.

### Stress resistance

**Fig 3:**
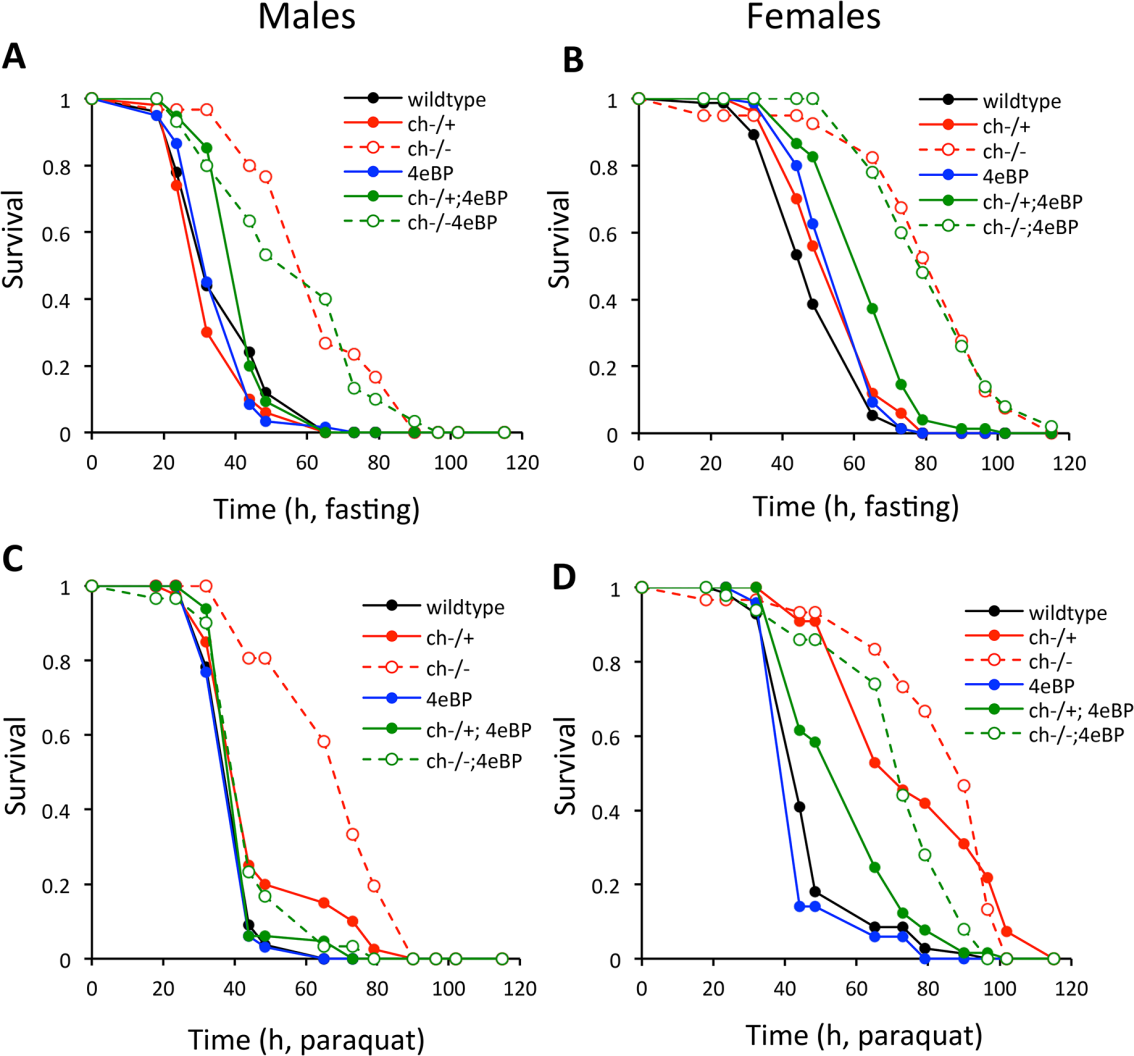
Resistance to acute stress-challenge by Drosophila with single and combined mutations of *chico* and *d4eBP*. Survival of adult **A)** males and **B)** females when fasting with water. Survival of adult **C)** males and **D)** females when exposed to paraquat.

Genes that confer longevity assurance often increase stress resistance. As has been previously documented for mutants of Drosophila insulin/IGF signaling including *chico* [2], *ch* ^-/-^ strongly extends fasting survival relative to wildtype and to *d4eBP* (Fig 3A and 3B) (Table 2). In contrast with earlier reports where loss of *d4eBP* reduces fasting survival [35,37], here *d4eBP* mutants alone modestly *increase* female fasting survival and is neutral in males. The improved fasting survival of *ch* ^-/-^ is modestly epistatic to the loss of *d4eBP* in double mutant females, but not so in *ch* ^-/-^ males. *Chico* heterozygotes again present different interactions (Fig 3A and 3B) (Table 2). *Chico* heterozygosity modestly increases fasting survival in females and not in males, but in males the loss of *d4eBP* in the *ch* ^+/-^ background synergistically increases fasting survival more than expected from the combined effects of single mutants.

**Table 2:** Proportional hazard analysis for survival during paraquat exposure and starvation. Proportional hazard modeled for *ch* ^*+/-*^ and *d4eBP* as single and double mutants relative to wildtype, and *ch* ^*-/-*^ and *d4eBP* as single and double mutants, with likelihood ratio test. Coefficient *β* for single loci: when significantly less than zero indicates reduction in mortality, estimates greater than zero indicate elevated mortality. Double mutants: coefficient *β* when significantly different from zero indicates gene interaction where the effect of the double mutant differs from expectation from product of single mutants. Epistasis inferred when significant gene interaction increases mortality (positive *β*) relative to expected product of *chico* and *d4eBP*; synergy inferred when gene interaction reduces mortality (negative *β*) relative to product of *chico* and *d4eBP* genotypes. Female and male survivorship when exposed to paraquat. Female and male survivorship during fasting.

	*β*	χ^2^	Prob.	Inference
**Paraquat females**				
*d4eBP*	0.343	29.32	< 0.0001	Increases mortality
*ch* ^*+/-*^	-0.448	38.04	< 0.0001	Reduces mortality
*ch* ^*-/-*^	-0.677	59.44	< 0.0001	Reduces mortality
*ch* ^*+/-*^ *d4eBP*	0.137	4.12	0.0423	Epistasis
*ch* ^*-/-*^ *d4eBP*	0.168	4.43	0.0354	Epistasis
**Paraquat males**				
*d4eBP*	0.0591	0.717	0.397	Not significant
*ch* ^*+/-*^	-0.147	4.313	0.0378	Reduces mortality
*ch* ^*-/-*^	-0.521	32.21	< 0.0001	Reduces mortality
*ch* ^*+/-*^ *d4eBP*	0.0459	0.433	0.510	No epistasis
*ch* ^*-/-*^ *d4eBP*	0.305	12.46	0.0004	Epistasis
**Fasting females**				
*d4eBP*	-0.243	10.16	0.0014	Reduces mortality
*ch* ^*+/-*^	-0.264	11.94	0.0005	Reduces mortality
*ch* ^*-/-*^	-0.978	107.7	< 0.0001	Reduces mortality
*ch* ^*+/-*^ *d4eBP*	0.0119	0.0212	0.884	No Epistasis
*ch* ^*-/-*^ *d4eBP*	0.177	5.81	0.0159	Epistasis
**Fasting males**				
*d4eBP*	-0.0477	0.582	0.445	Not significant
*ch* ^*+/-*^	-0.0299	0.203	0.652	Not significant
*ch* ^*-/-*^	-0.626	46.66	< 0.0001	Reduces mortality
*ch* ^*+/-*^ *d4eBP*	-0174	6.653	0.0099	Synergy
*ch* ^*-/-*^ *d4eBP*	0.00599	0.0054	0.941	No epistasis

Resistance to paraquat, a generator of oxidative stress, is elevated in both *ch* ^+/-^ and *ch* ^-/-^ females, and both *chico* genotypes modestly require *d4eBP* for this benefit (Fig 3C and 3D) (Table 2). In males, *ch* ^-/-^ produces a large benefit upon paraquat challenge that largely requires *d4eBP*. Male *ch* ^+/-^ produce a small survival benefit and provide no evidence for genetic interaction with *d4eBP*. Overall, resistance to paraquat is improved by both genotypes of *chico* and this requires 4E-BP.

### Size

**Fig 4:**
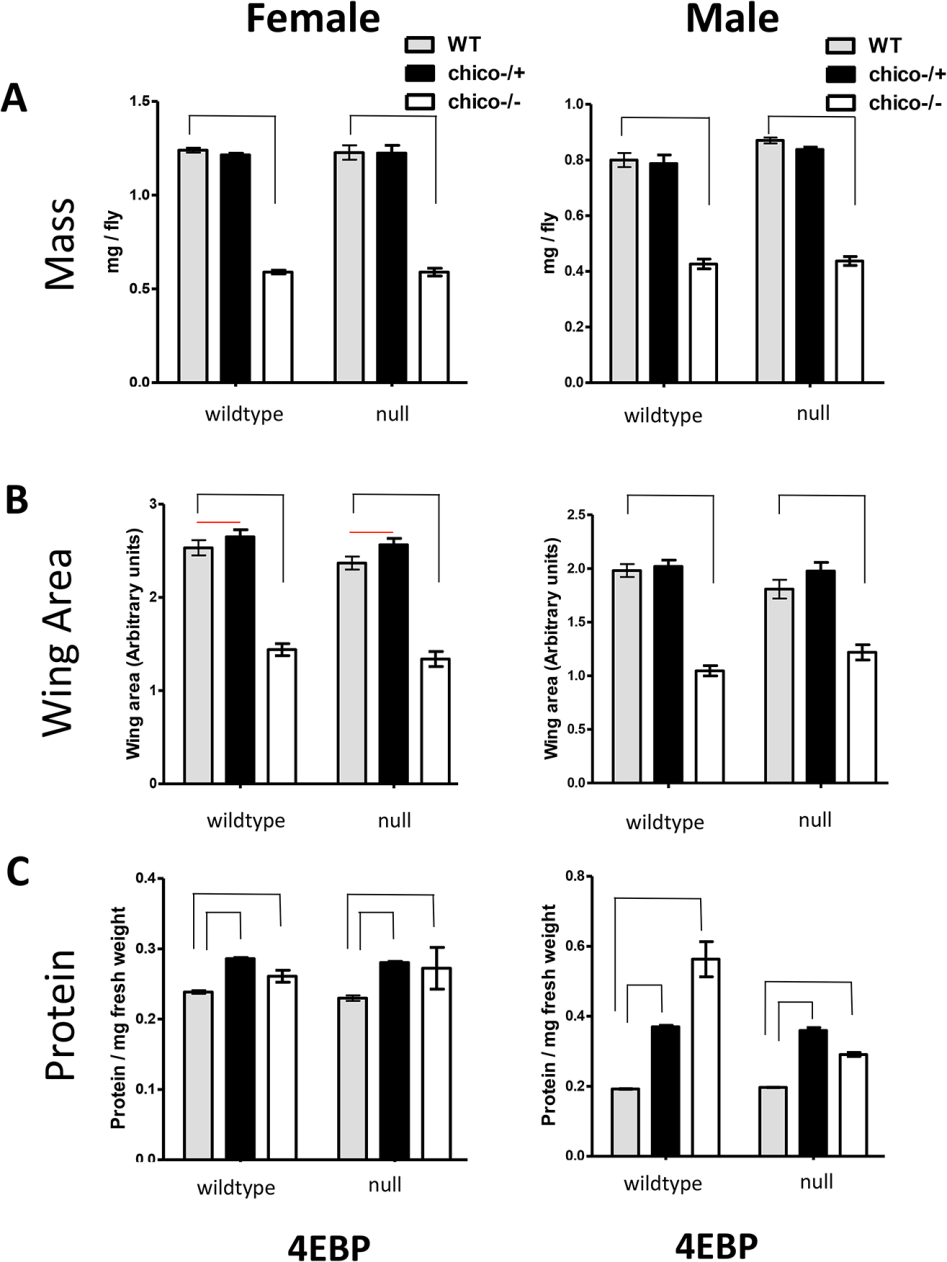
Impact of *chico* and *d4eBP* alone and together upon measures of adult body size. Females in left columns, males in right columns. Within each panel, *chico* genotypes are plotted for categories of *d4eBP* (wildtype and null mutant). A) Fresh body mass. B) Total wing area. C) Protein content per fresh body mass. Statistically significant differences among *chico* genotypes within *d4eBP* genotypes are indicated by brace-lines: p < 0.05. Statistics for interaction effects between genotypes are described in main text.

*Chico* homozygotes are diminutive (Fig 4). In female *ch* ^-/-^, the *d4eBP* mutant alone did not affect mass or wing area (ANOVA two-way interaction, p > 0.53). In males, the *d4eBP* mutant slightly reduced the impact of *ch* ^-/-^ upon wing area (p = 0.016) but not upon mass. Protein content was marginally elevated in *ch* ^-/-^ females and independent of *d4eBP* (p = 0.529), but strongly so in males and in a *d4eBP*-dependent manner (p < 0.0001). Thus, while growth is affected by *ch* ^-/-^, these traits are independent of 4E-BP in females but somewhat dependent on 4E-BP in males.

As noted, size is not a determinant of longevity assurance since the long-lived *chico* heterozygotes are no smaller than wildtype. In fact, the wing area of female *ch* ^+/-^, but not males, was slightly larger than wildtype (Fig 4B). No interaction between *ch* ^+/-^ and *d4eBP* was detected in either sex (ANOVA two-way interaction p > 0.38). Mass of *ch* ^+/-^ did not differ from wildtype in either sex, or depend on *d4eBP* (p > 0.63). Protein content was elevated in *ch* ^+/-^ males and females (Fig 4C), but again independent of *d4eBP* (p > 0.14).

### Signaling

**Fig 5:**
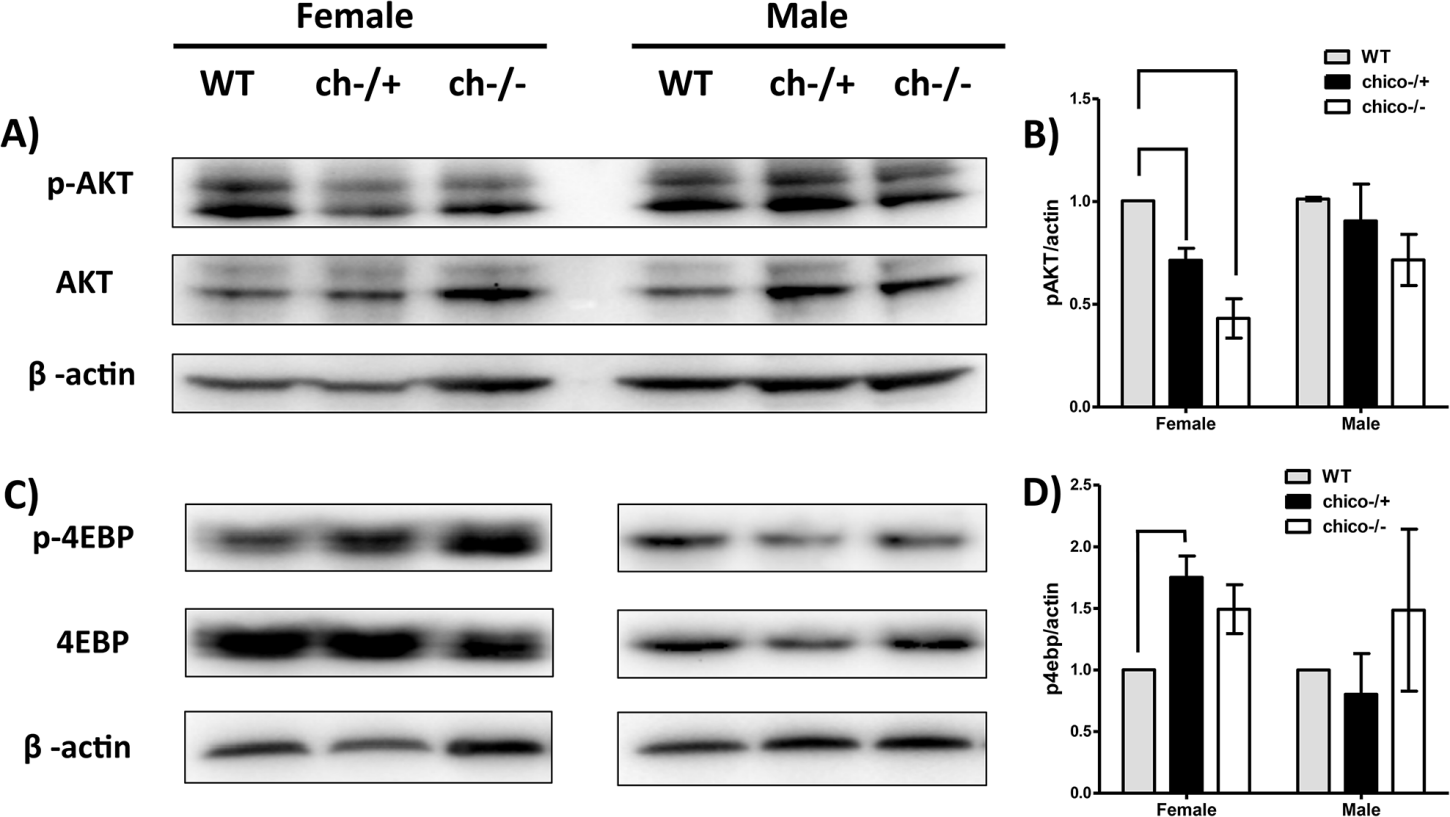
Abundance of AKT, pAKT, 4E-BP and p4E-BP in whole adults of each *chico* genotype. **A)** and **C)** Western blots with actin loading control. **B)** and **D)** Means (s.e.) from quantified replicate blots (n = 3) relative to actin within each matched sample. Significant differences (ANOVA, post hoc test) indicated by brace-lines: p < 0.05.

Drosophila insulin receptor substrate CHICO recruits and activates PI3K (p110) at the cell membrane, which in turn acts with PtdIns(3,4,5)*P*_3_ to recruit and phosphorylate AKT [40,41]. *Chico* homozygotes were reported to interact with mutants of the insulin receptor locus (*InR*) to control growth [31], and further genetic study between dPKB (AKT) and dPTEN (p110 PI3K) loss-of-function mutants implicated Drosophila AKT as the major mediator of PI3K control of growth [42,43]. Here, consistent with observations for larvae [44], AKT phosphorylation is reduced in females for both *chico* genotypes, while only a small, non-significant reduction in pAKT is observed for males (Fig 5A and 5B). In cell culture, pAKT phosphorylates and inactivates tuberous sclerosis complex 2, thereby preventing TSC1/2 repression of TORC1 and permitting this kinase to phosphorylate and inactive 4E-BP. As noted, the relevance of this activity of AKT upon TORC1 is debated in the context of Drosophila physiology [12]. Pallares-Cartes [45] demonstrated that *chico* ^*1*^ homozygotes, via PRAS40, modulate TORC1 only within ovarian tissue and not in somatic tissue. Here, from whole females, we observe that the level of 4E-BP phosphorylation (inactive state) is *increased* (rather than decreased) in all *chico* genotypes, and significantly so in *ch* ^*+/-*^ (Fig 5C and 5D). In males we detect no significant differences in p4E-BP between wildtype and either *chico* genotype. Contrary to expectation, the inactive p4E-BP form is not decreased in animal tissue of *chico* mutant Drosophila.

## Discussion

These results reveal a complex of unexpected outcomes (summarized in S1 Table). Neither *chico* genotype decreases the phosphorylation of 4E-BP. This observation contrasts with results for how insulin/IGF signaling impacts TOR in cell culture [19,46] but not with *in vivo* analyses of Drosophila somatic tissue of *chico* homozygotes [45,47–49]. Furthermore, *ch* ^+/-^ actually elevates p4E-BP sampled from whole *ch* ^*+/-*^ females, suggesting these adults have *elevated* rather than repressed TORC1 activity.

Secondly, *chico* heterozygotes and homozygotes appear to regulate aging in different ways. Retarded demographic aging by *ch* ^-/-^ does not require *d4eBP*. Although *ch* ^-/-^ reduces pAKT, whether *foxo* is required for *ch* ^-/-^ to extend lifespan has not been reported to date, and it remains an open question as to what downstream pathways are required for *ch* ^-/-^ to slow aging. On the other hand, previous reports show that *ch* ^+/-^ requires *foxo* to fully extend lifespan [16,17], and that *ch* ^+/-^ in part modulates longevity assurance through activin signaling that is repressed by *chico*/FOXO [50]. Here we see that *ch* ^+/-^ also partially requires *d4eBP* to slow demographic and functional aging.

Strikingly, *ch* ^+/-^ females have *increased* fecundity. This is somewhat unexpected since insulin/IGF signaling drives germline stem cell maintenance, proliferation, and niche organization, but could be explained if *ch* ^+/-^ increases TORC1 activity and thus increases pS6K [51–53]. In a further twist, *d4eBP*-null increases fecundity in the youngest females and independently of *chico*. These reproductive effects contrast with *chico* homozygotes, which are completely sterile. Besides regulating aging in different ways, *chico* genotypes appear to differentially modulate reproduction.

Thirdly, while *ch* ^+/-^ partially requires *d4eBP* to slow aging, at least in females this *chico* genotype increases the *inactive* form of the translation inhibitor (p4E-BP). 4E-BP binds and sequesters eIF4E away from eIF4G to inhibit cap-dependent translation, while phosphorylated binding protein (p4E-BP) disassociates from eIF4G to permit formation of the active translational complex [54–56]. We note that substantial non-phosphorylated 4E-BP remains in *chico* heterozygotes. Based on our genetic data we propose that CHICO can interact (directly or indirectly) with this residual, active 4E-BP to regulate longevity assurance. In this model, *ch* ^+/-^ changes some quality of insulin/IGF signaling to amplify the impact of the residual, active 4E-BP. This idea may explain why ectopic expression of constitutively active 4E-BP increases Drosophila survival [23], and that rapamycin fed to long-lived IIS mutants of Drosophila is still able to extend lifespan [26]. Similarly, expression of activated 4E-BP1 in mouse skeletal muscle protects against age-related decline in insulin-sensitivity and metabolic rate, apparently through muscle autonomous increase in translation of peroxisome-proliferation-activated receptor-**γ** coactivator-1α (PGC-1 α) [57].

This model assumes that *ch* ^+/-^ can regulate 4E-BP activity independent of TORC1 (S4 Fig). Drosophila 4E-BP interacts with eIF4E through canonical and non-canonical binding motifs, which permits even low concentrations of 4E-BP to efficiently compete against eIF4G [58]. While TORC1-independent interactions between IRS and 4E-BP are currently unknown, we can entertain several possibilities. *Chico* heterozygotes may increase the duration of 4E-BP binding with eIF4E in mRNP (messenger ribonucleoprotein) granules and thus reinforce translation repression [59]. Alternatively, downstream consequences of translation repression might interact with effects of FOXO-mediated transcription. Finally, 4E-BP typically functions in the cytoplasm [60], but some of this protein interacts with eIF4E in mammalian nuclei [61]. While the function of nuclear 4E-BP is unknown, it might be sensitive to IRS. Although these suggestions are speculative, they reflect a need to explore ways IRS can affect 4E-BP function through mechanisms that do not involve its presumed control through PI3K/AKT/TSC/TORC1.

## Supporting Information

S1 Fig Cross scheme for generation of *d4eBP*, *chico* double mutants. (PPTX)

S2 Fig Location of PCR primers for detecting *d4eBP/Thor* deletion. (PPTX)

S3 Fig Identifying heterozygous and homozygous *Thor* mutants via PCR. (PPTX)

S4 Fig Graphical model for action of Chico pathways affecting aging. (PPTX)

S1 Table Summarizes results across all tests, genotypes and sex. (DOCX)

S1 Text Supplemental Experimental Procedures. (DOCX)
