# Supplementary material for: Drosophila Longevity Assurance Conferred by Reduced Insulin Receptor Substrate Chico Partially Requires d4eBP
Source: PLoS One. 2015 Aug 7;10(8):e0134415. doi: 10.1371/journal.pone.0134415 (PMC4529185; doi:10.1371/journal.pone.0134415)
Supplement: S1 Fig — (PPTX) [file pone.0134415.s001.pptx]

## Slide 1
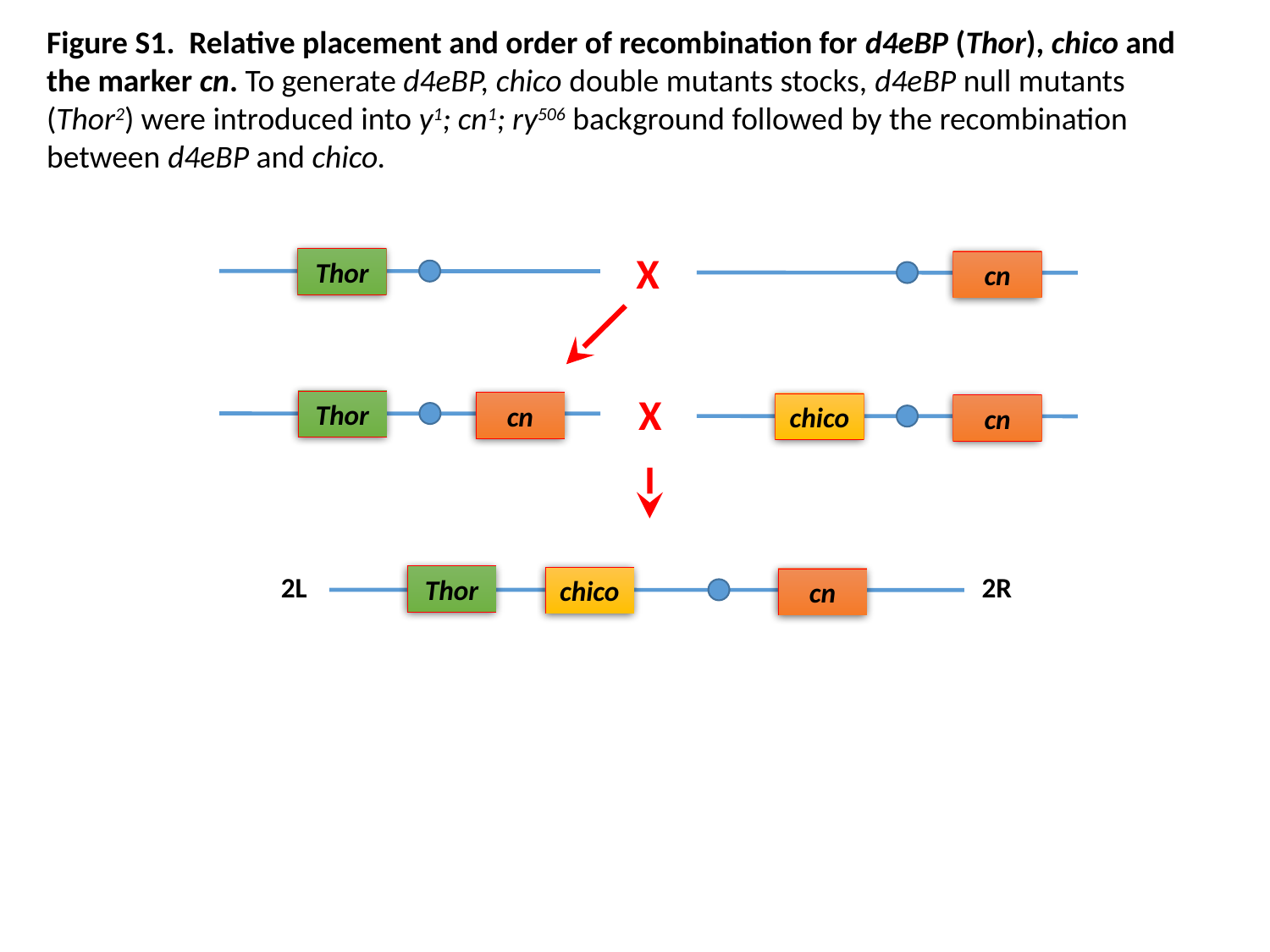

Figure S1. Relative placement and order of recombination for d4eBP (Thor), chico and the marker cn. To generate d4eBP, chico double mutants stocks, d4eBP null mutants (Thor2) were introduced into y1; cn1; ry506 background followed by the recombination between d4eBP and chico.
X
Thor
cn
X
Thor
cn
chico
cn
2R
2L
Thor
chico
cn
