# Supplementary material for: Drosophila Longevity Assurance Conferred by Reduced Insulin Receptor Substrate Chico Partially Requires d4eBP
Source: PLoS One. 2015 Aug 7;10(8):e0134415. doi: 10.1371/journal.pone.0134415 (PMC4529185; doi:10.1371/journal.pone.0134415)
Supplement: S2 Fig — (PPTX) [file pone.0134415.s002.pptx]

## Slide 1
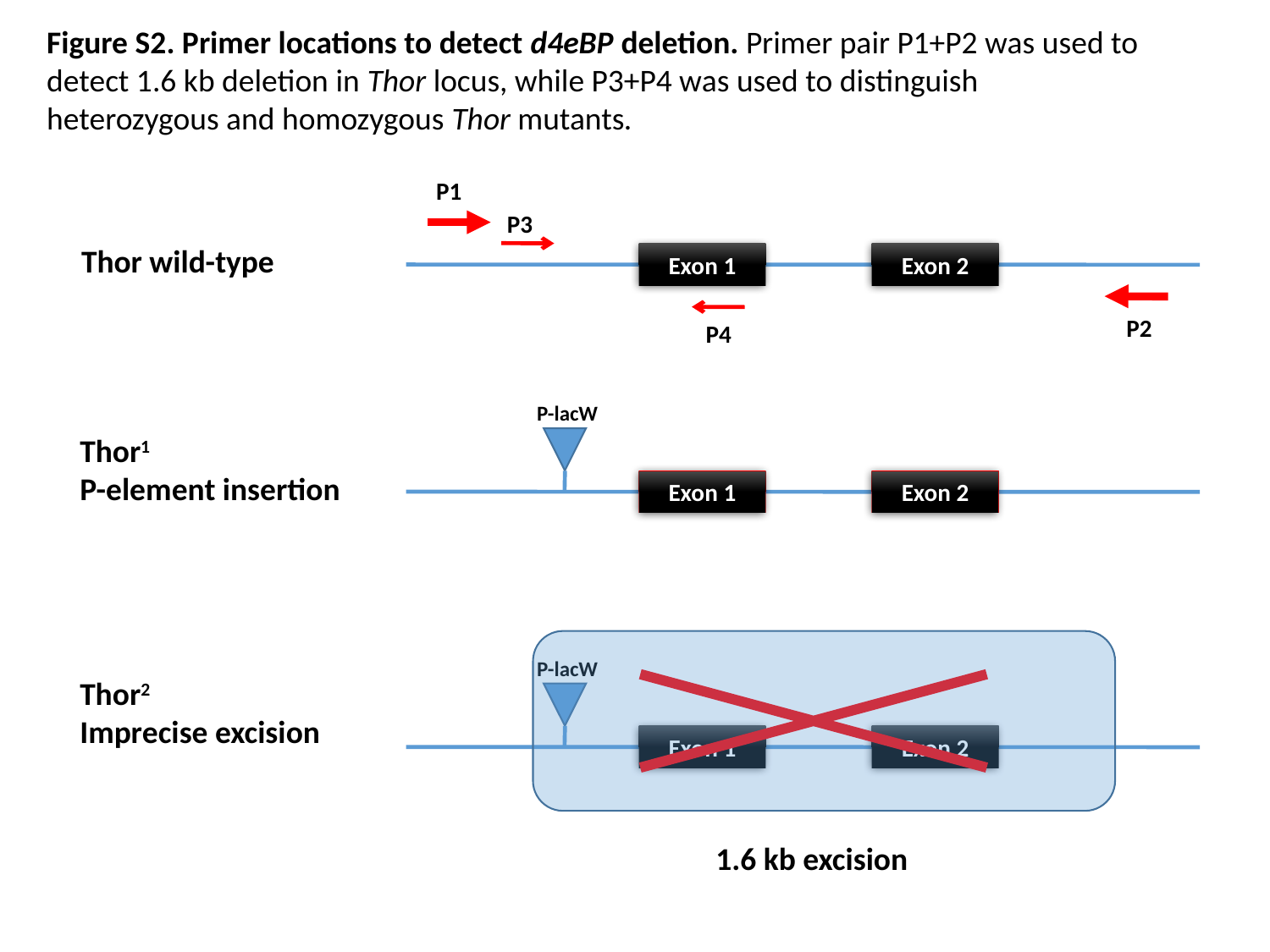

Figure S2. Primer locations to detect d4eBP deletion. Primer pair P1+P2 was used to detect 1.6 kb deletion in Thor locus, while P3+P4 was used to distinguish heterozygous and homozygous Thor mutants.
P1
P3
Thor wild-type
Exon 1
Exon 2
P2
P4
P-lacW
Exon 1
Exon 2
Thor1
P-element insertion
P-lacW
Exon 1
Exon 2
Thor2
Imprecise excision
1.6 kb excision
