# Supplementary material for: Drosophila Longevity Assurance Conferred by Reduced Insulin Receptor Substrate Chico Partially Requires d4eBP
Source: PLoS One. 2015 Aug 7;10(8):e0134415. doi: 10.1371/journal.pone.0134415 (PMC4529185; doi:10.1371/journal.pone.0134415)
Supplement: S3 Fig — (PPTX) [file pone.0134415.s003.pptx]

## Slide 1
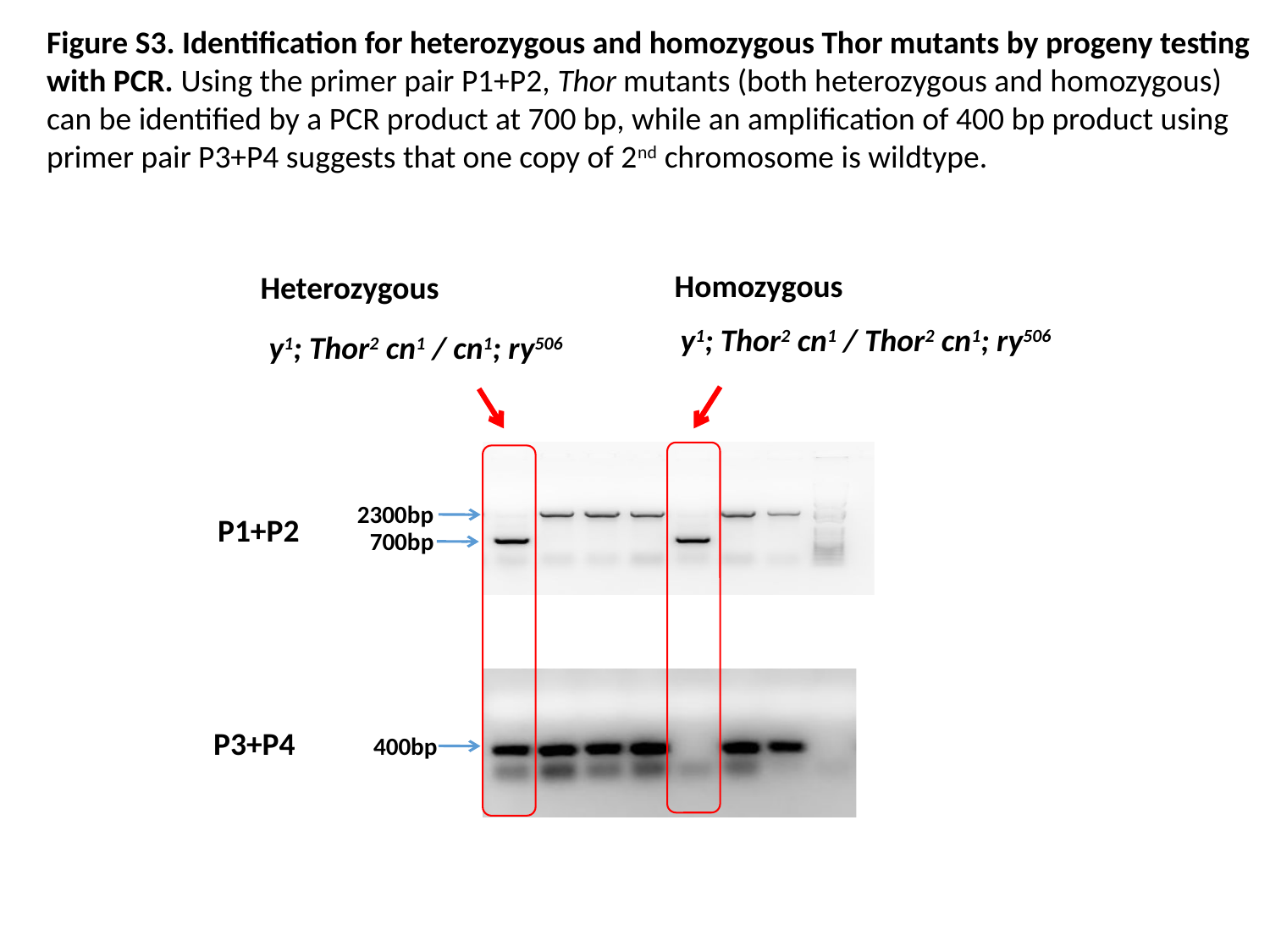

Figure S3. Identification for heterozygous and homozygous Thor mutants by progeny testing with PCR. Using the primer pair P1+P2, Thor mutants (both heterozygous and homozygous) can be identified by a PCR product at 700 bp, while an amplification of 400 bp product using primer pair P3+P4 suggests that one copy of 2nd chromosome is wildtype.
Homozygous
y1; Thor2 cn1 / Thor2 cn1; ry506
Heterozygous
y1; Thor2 cn1 / cn1; ry506
2300bp
P1+P2
700bp
P3+P4
400bp
