# Supplementary material for: Drosophila Longevity Assurance Conferred by Reduced Insulin Receptor Substrate Chico Partially Requires d4eBP
Source: PLoS One. 2015 Aug 7;10(8):e0134415. doi: 10.1371/journal.pone.0134415 (PMC4529185; doi:10.1371/journal.pone.0134415)
Supplement: S4 Fig — (PPTX) [file pone.0134415.s004.pptx]

## Slide 1
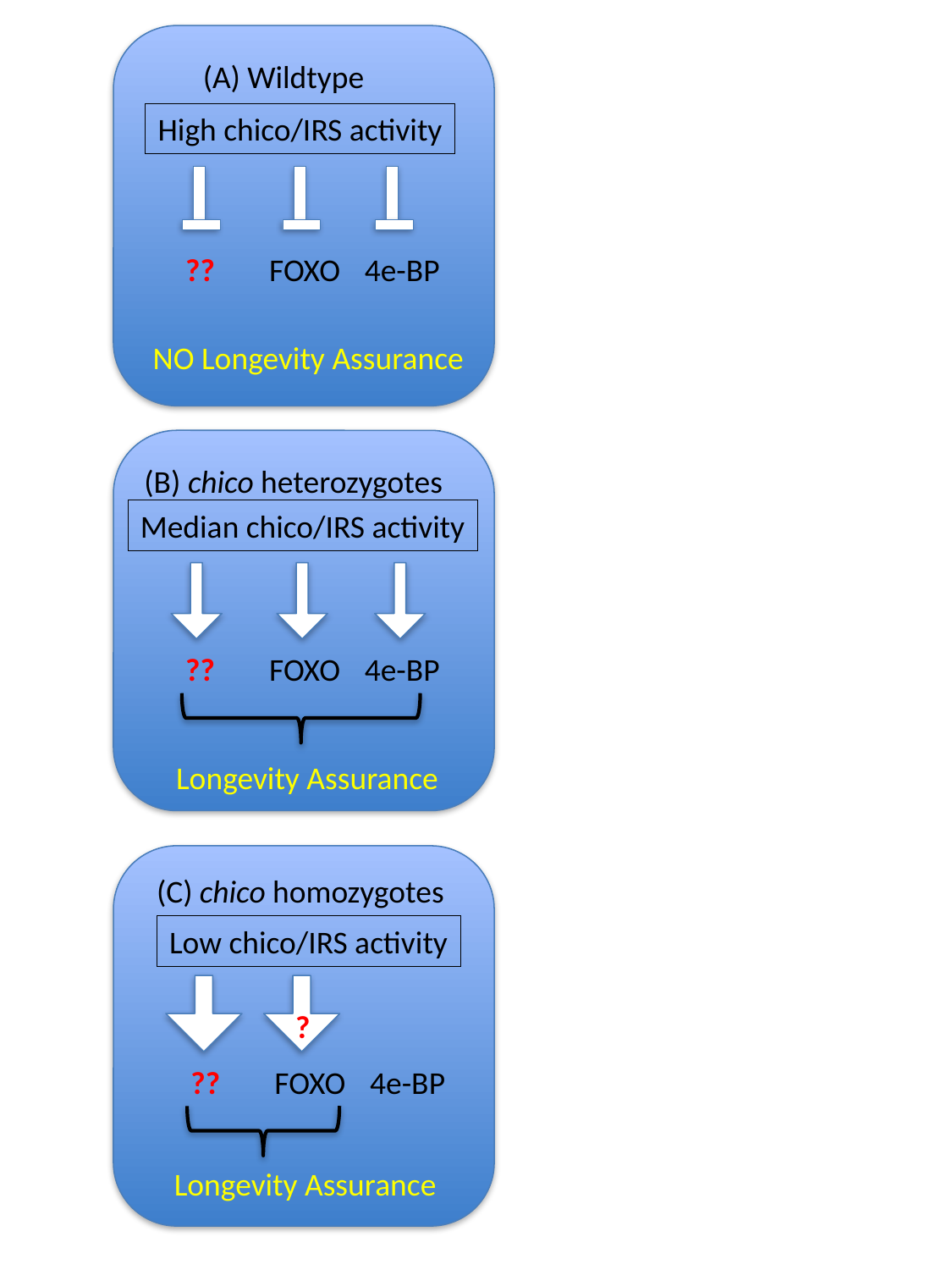

(A) Wildtype
High chico/IRS activity
??
FOXO
4e-BP
NO Longevity Assurance
(B) chico heterozygotes
Median chico/IRS activity
??
FOXO
4e-BP
Longevity Assurance
(C) chico homozygotes
Low chico/IRS activity
??
FOXO
4e-BP
?
Longevity Assurance
