# Supplementary material for: Drosophila Longevity Assurance Conferred by Reduced Insulin Receptor Substrate Chico Partially Requires d4eBP
Source: PLoS One. 2015 Aug 7;10(8):e0134415. doi: 10.1371/journal.pone.0134415 (PMC4529185; doi:10.1371/journal.pone.0134415)
Supplement: S1 Table — (DOCX) [file pone.0134415.s005.docx]

|  | Female | | Male | |
| --- | --- | --- | --- | --- |
| Trait: | *Chico+/-* | *Chico-/-* | *Chico+/-* | *Chico-/-* |
| *Age-dependent:* | | | | |
| Mortality | **YES** | **NO** | **YES** | **NO** |
| Fecundity | **NO** | *NA* | *NA* | *NA* |
| Climbing | **YES** | **NO** | *NA* | *NA* |
| *Size and body composition:* | | | | |
| Mass | **NO** | **NO** | **NO** | **NO** |
| Wing length | **NO** | **NO** | **NO** | **YES** |
| Protein | **NO** | **NO** | **NO** | **YES** |
| *Stress resistance:* | | | | |
| Starvation | **NO** | **YES** | **YES** | **NO** |
| Paraquat | **YES** | **YES** | **YES** | **YES** |

**Summary of genetic interactions (epistasis) between *chico* genotypes and *d4eBP*.**

*NA*: data not available
