# Supplementary material for: Drosophila Longevity Assurance Conferred by Reduced Insulin Receptor Substrate Chico Partially Requires d4eBP
Source: PLoS One. 2015 Aug 7;10(8):e0134415. doi: 10.1371/journal.pone.0134415 (PMC4529185; doi:10.1371/journal.pone.0134415)
Supplement: S1 Text — (DOCX) [file pone.0134415.s006.docx]

Drosophila longevity assurance conferred by reduced Insulin Receptor Substrate *chico* partially requires 4E-BP

Hua Bai^1^, Stephanie Post^1^, Ping Kang^1^, Marc Tatar^1*^

^1^Department of Ecology and Evolutionary Biology, Division of Biology and Medicine, Brown University, Providence, Rhode Island, USA

* Corresponding author

Email: Marc_Tatar@Brown.edu

**S1. Supplemental Experimental Procedures**

**1.1. Generation of *d4eBP, chico* double mutants**

**(A). Introducing *d4eBP* null mutants (*Thor^2^*) into *y^1^; cn^1^; ry^506^*, the *chico* segregating stock background.** The *chico* segregating stock (*y^1^; chico^1^ cn^1^ / cn^1^; ry^506^*) was previously generated in our laboratory by introducing *chico^1^* into the base stock *y^1^; cn^1^; ry^506^* (Tu M-P, Epstein D, Tatar M. The demography of slow aging in male and female Drosophila mutant for the insulin-receptor substrate homolog chico. Aging Cell. 2002;1: 75-80). This segregating stock is maintained without balancers by breading heterozygotes. All three genotypes segregate among offspring in each generation and are distinguished by eye color and body size.

To generate *d4eBP, chico* double mutants, we first introduced *d4eBP* mutants (*Thor^2^*) into our base stock *y^1^; cn^1^; ry^506^* (S3 Figure). Since the *d4eBP* null mutant (*thor^2^*) was produced by imprecise mobilization of a P-element insertion at the *Thor* locus (located on 2^nd^ chromosome) and this provides no visible marker available to monitor the deletion, we designed two pairs of PCR primers to detect the mutations (S4 Figure). The primers used for detecting the deletion are P1 (AGATAAACAAGAGCTCAAGGCG) and P2 (ACTCTGTAAGCATCTGGGATCC). To distinguish homozygous from heterozygous mutants, we designed another pair of primers (P3: GCAGAATAATCAGGCGAGAG, P4: TCGAAACTTACCTCCAGGAG), which can only amplify the *Thor* locus in wild-type chromosome, but not the mutated one (S5 Figure).

Initially, we outcrossed the original *Thor* null mutants *yw; Thor^2^; +* (males) to *y^1^; cn^1^; ry^506^* (virgin females). The male F1 of this cross (*y^1^; Thor^2^ / cn^1^; ry^506^/+*) were backcrossed to *y^1^; cn^1^; ry^506^* (virgin females). Virgin offspring females from this cross that carry *Thor^2^ cn^1^* recombination (*y^1^; Thor^2^ cn^1^ / cn^1^; ry^506^/+*) were individually backcrossed to *y^1^; cn^1^; ry^506^* males for 6 generations. In each backcross, female parents were sacrificed after egg laying and genomic DNA were extracted for PCR verification of the present of *Thor* mutation. To generate *Thor^2^* homozygous recombination lines, we performed more than 100 individual crosses from the backcrossed stocks (a mixture of two genotypes: *y^1^; cn^1^ / cn^1^; ry^506^/ ry^506^* and *y^1^; Thor^2^ cn^1^ / cn^1^; ry^506^/ ry^506^*, both have apricot eyes). Again, PCR was used to detect the present of *Thor^2^* hetero- and homozygous. We were able to recover two independent recombinant lines that were homozygous for *Thor^2^* (*y^1^; Thor^2^ cn^1^ / Thor^2^ cn^1^; ry^506^/ ry^506^*).

**(B). Recombination between *d4eBP/Thor* and *chico***. We first crossed *y^1^; Thor^2^ cn^1^ / Thor^2^ cn^1^; ry^506^/ ry^506^* (virgin females) to *y^1^; chico^1^ cn^1^ / cn^1^; ry^506^ / ry^506^* (males) to generate stocks *y^1^; chico^1^ cn^1^ / Thor^2^ cn^1^; ry^506^ / ry^506^* (cinnabar eyes). Next, recombination between *Thor^2^* and *chico^1^* was conducted by crossing *y^1^; chico^1^ cn^1^ / Thor^2^ cn^1^; ry^506^ / ry^506^* (virgin females) to *y^1^; chico^1^ cn^1^ / cn^1^; ry^506^ / ry^506^* (males). Male F1 flies with cinnabar eye color and normal body size, a mixture of three genotypes (*y^1^; Thor^2^ chico^1^ cn^1^ / cn^1^; ry^506^ / ry^506^*; *y^1^; chico^1^ cn^1^ / Thor^2^ cn^1^; ry^506^ / ry^506^*; *y^1^; chico^1^ cn^1^ / cn^1^; ry^506^ / ry^506^*), were individually crossed to virgin female of *y^1^; chico^1^ cn^1^ / cn^1^; ry^506^ / ry^506^*. After mating, male parents were sacrificed and tested the present of *Thor^2^* mutation by genomic PCR methods. The recombination event in the F1 was further verified by examining the genotypes of the F2 flies. The presence of *y^1^; cn^1^ / cn^1^; ry^506^ / ry^506^* and the absence of *y^1^; Thor^2^ cn^1^ / cn^1^; ry^506^ / ry^506^* indicate that the male parents contain the recombination between *Thor^2^* and *chico^1^* (*y^1^; Thor^2^ chico^1^ cn^1^ / cn^1^; ry^506^ / ry^506^*).

**(C). Generation of stable *d4eBP/Thor* and *chico* double mutant stocks***.* We crossed *y^1^; Thor^2^ cn^1^ / Thor^2^ cn^1^; ry^506^/ ry^506^* (virgin females) and *y^1^; Thor^2^ chico^1^ cn^1^ / cn^1^; ry^506^ / ry^506^* (males). PCR and eye color were used to identify *y^1^; Thor^2^ chico^1^cn^1^ / Thor^2^ cn^1^; ry^506^/ ry^506^* from the F1 progeny. Selected F1 males were again backcrossed *Thor^2^* stocks (*y^1^; Thor^2^ cn^1^ / Thor^2^ cn^1^; ry^506^/ ry^506^*) to generate stable double mutant stocks (*y^1^; Thor^2^ chico^1^cn^1^ / Thor^2^ cn^1^; ry^506^/ ry^506^*).

**1.2. Demography**

Six otherwise coisogenic genotypes with single *chico* and *Thor^2^* mutations and double mutant stocks were used in demographic studies:

|  | Genotype | Phenotype |
| --- | --- | --- |
| *wt* | *y^1^; cn^1^ / cn^1^; ry^506^ / ry^506^* | Apricot eyes, normal body size |
| *ch +/-* | *y^1^; chico^1^ cn^1^ / cn^1^; ry^506^ / ry^506^* | Cinnabar eyes, normal body size |
| *ch -/-* | *y^1^; chico^1^ cn^1^ / chico^1^ cn^1^; ry^506^ / ry^506^* | Cinnabar eyes, small body size |
| *4eBP* | *y^1^; Thor^2^ cn^1^ / Thor^2^ cn^1^; ry^506^ / ry^506^* | Apricot eyes, normal body size |
| *4eBP,ch +/-* | *y^1^; Thor^2^ chico^1^ cn^1^ / Thor^2^ cn^1^; ry^506^ / ry^506^* | Cinnabar eyes, normal body size |
| *4eBP,ch -/-* | *y^1^; Thor^2^ chico^1^ cn^1^ / Thor^2^ chico^1^ cn^1^; ry^506^ / ry^506^* | Cinnabar eyes, small body size |

Cohorts for each genotype were collected after eclosion, and females and males are separated after two days mating and transferred to demography cages at a density of 125 flies per cage. Three independent cages were set-up per genotype. Two independent demographic trials were performed with 125 adult flies per demography cage and three replicate cages were per genotype.
